# A Serum Protein Biomarker Panel Improves Outcome Prediction in Human Traumatic Brain Injury

**DOI:** 10.1089/neu.2019.6375

**Published:** 2019-09-23

**Authors:** Eric Thelin, Faiez Al Nimer, Arvid Frostell, Henrik Zetterberg, Kaj Blennow, Harriet Nyström, Mikael Svensson, Bo-Michael Bellander, Fredrik Piehl, David W. Nelson

**Affiliations:** ^1^ Department of Clinical Neuroscience, Karolinska Institutet, Stockholm, Sweden.; ^2^ Department of Neurology, Karolinska University Hospital, Stockholm, Sweden.; ^3^ Division of Neurosurgery, Department of Clinical Neurosciences, University of Cambridge, Cambridge, United Kingdom.; ^4^ Clinical Neurochemistry Laboratory, Sahlgrenska University Hospital, Mölndal, Sweden.; ^5^ Department of Psychiatry and Neurochemistry, Institute of Neuroscience and Physiology, the Sahlgrenska Academy at the University of Gothenburg, Mölndal, Sweden.; ^6^ Department of Neurodegenerative Disease, UCL Institute of Neurology, Queen Square, London, United Kingdom.; ^7^ UK Dementia Research Institute, UCL, London, United Kingdom.; ^8^ Department of Neuroradiology, Karolinska University Hospital, Stockholm, Sweden.; ^9^ Department of Neurosurgery, Karolinska University Hospital, Stockholm, Sweden.; ^10^ Department of Physiology and Pharmacology, Section of Perioperative Medicine and Intensive Care, Karolinska Institutet, Stockholm, Sweden.

**Keywords:** functional outcome, injury severity assessment, neuroradiology, protein biomarkers, serum analysis, traumatic brain injury

## Abstract

Brain-enriched protein biomarkers of tissue fate are being introduced clinically to aid in traumatic brain injury (TBI) management. The aim of this study was to determine how concentrations of six different protein biomarkers, measured in samples collected during the first weeks after TBI, relate to injury severity and outcome. We included neurocritical care TBI patients that were prospectively enrolled from 2007 to 2013, all having one to three blood samples drawn during the first 2 weeks. The biomarkers analyzed were S100 calcium-binding protein B (S100B), neuron-specific enolase (NSE), glial fibrillary acidic protein (GFAP), ubiquitin carboxy-terminal hydrolase-L1 (UCH-L1), tau, and neurofilament-light (NF-L). Glasgow Outcome Score (GOS) was assessed at 12 months. In total, 172 patients were included. All serum markers were associated with injury severity as classified on computed tomography scans at admission. Almost all biomarkers outperformed other known outcome predictors with higher levels the first 5 days, correlating with unfavorable outcomes, and UCH-L1 (0.260, pseduo-*R*^2^) displaying the best discrimination in univariate analyses. After adjusting for acknowledged TBI outcome predictors, GFAP and NF-L added most independent information to predict favorable/unfavorable GOS, improving the model from 0.38 to 0.51 pseudo-*R*^2^. A correlation matrix indicated substantial covariance, with the strongest correlation between UCH-L1, GFAP, and tau (*r* = 0.827–0.880). Additionally, the principal component analysis exhibited clustering of UCH-L1 and tau, as well as GFAP, S100B, and NSE, which was separate from NF-L. In summary, a panel of several different protein biomarkers, all associated with injury severity, with different cellular origin and temporal trajectories, improve outcome prediction models.

## Introduction

Traumatic brain injury (TBI) is a devastating disease and one of the most common reasons people are living with acquired disabilities,^1^ leading to increasing suffering and societal costs. TBI usually results in a combination of diffuse tissue injuries and a spectrum of focal lesions, as well as a range of subsequent secondary injury responses, making TBI a biologically very complex and heterogenic condition.^2^ Analyzing admission parameters have improved outcome prediction in TBI,^3,4^ which may provide tools for resource allocation both on the group level, but also on individual treatment strategies. However, the performance of current prediction models is limited and much variance remains unexplained.^5,6^

Serum brain-enriched proteins of tissue fate are increasingly used as biomarkers to manage TBI patients.^7^ For example, serum S100 calcium-binding protein B (S100B) is part of the Scandinavian neurotrauma guidelines to reduce the number of unnecessary computerized tomography (CT) scans in mild TBI patients.^8^ Biomarkers may also be used to monitor emerging secondary injury processes, as well as to improve outcome prediction.^9–11^ Whereas these biomarkers are often used as single variables, by analyzing several proteins, with different cellular origins, it may be possible to delineate distinct pathophysiological processes in the injured brain,^12^ which, in turn, could result in more-precise outcome predictions.

The most well-studied protein in TBI is S100B, with a predominantly astrocytic origin, which has been shown to be a robust, independent outcome predictor in TBI.^11^ Neuron-specific enolase (NSE) is a neuronal enriched glycolytic protein and is used in guidelines for cardiac arrest.^13^ Neurofilament-light (NF-L) is one of the main proteins of the neuroaxonal skeleton and is among the most promising biomarkers for disease severity in multiple sclerosis and amyotrophic lateral sclerosis,^14^ as well as in both mild and severe TBI.^15,16^ Serum levels of other brain-enriched proteins that have been suggested to predict outcome of TBI include glial fibrillary acidic protein (GFAP), an astrocytic cytoskeletal protein,^17^ and ubiquitin carboxy-terminal hydrolase-L1 (UCH-L1), a protein enriched in neurons involved in the production of ubiquitin.^18^ GFAP and UCH-L1 in tandem have recently been suggested to aid in screening mild TBI patients to avoid unnecessary CT scans.^19^ Other studied biomarkers include microtubule-associated protein tau, which is predominantly present in neurons and used clinically as a biomarker in Alzheimer's disease,^20^ but also in acute TBI as well as being aggregated in chronic traumatic encephalopathy.^21–23^

Apart from absolute levels, these proteins exhibit different temporal dynamics in serum.^6,24^ In comparison, UCH-L1, S100B, and tau seem to display short effective half-lives (hours) in blood as compared to GFAP and NSE (days), and for NF-L up to several weeks.^25,26^ Another important factor is to what degree a biomarker is brain enriched, given that contribution from extracranial injuries will affect specificity. For instance, S100B can be released from non-cerebral tissues in the early phase after injury,^6^ whereas GFAP and NF-L are virtually restricted to the nervous system.^27^ Collectively, to be able to accurately associate a biomarker concentration with outcome, it is of importance to determine the temporal profile of the biomarkers in relation to timing of injury, as well as to account for extracranial sources.

Previous studies addressing the predictive power of combinations of protein biomarkers have shown mixed results.^6,28–31^ However, many studies have been relatively small in terms of the number of included patients and analyzed markers. Additionally, few studies have adjusted for temporal dynamics and other acknowledged outcome predictors, such as the independent outcome variables in International Mission for Prognosis and Analysis of Clinical Trials in TBI (IMPACT).^3^

We aimed to assess the predictive power of a panel of six candidate TBI markers (S100B, NSE, NF-L, GFAP, UCH-L1, and tau) in serum, both independently and combined, while adjusting for known outcome predictors in a prospective cohort of neurocritical care unit (NCCU) TBI patients. As a secondary aim, we wished to establish the temporal profiles of these biomarkers, covariance between biomarkers, and association with intra- and extracranial injuries, in order to provide better outcome prediction models and understanding of the relative value of these biomarkers.

## Methods

### Study design, ethics, and setting

The data were collected as part of a prospective, observational study. TBI patients, 18–75 years age and admitted to the NCCU at the Karolinska University Hospital between 2007 and 2013, were enrolled. Ethical approval was provided by the regional ethical board in Stockholm (#2005/1526/31/2), and consent was provided by next of kin, in line with the Declaration of Helsinki. The study aimed to follow the STROBE statement for cohort studies (Supplementary Document S1).

### Traumatic brain injury management

TBI management at our department has been described in detail in previous studies.^6,29^ In short, we adhere to guidelines similar to that of the Brain Trauma Foundation.^32^ The upper intracranial pressure (ICP) threshold is 20 mm Hg, and in patients approaching the upper ICP limit, we aim for a cerebral perfusion pressure of 50–70 mm Hg. In patients with refractory high ICP, barbiturate coma (monitored and limited by burst suppression on electroencephalogram) was induced or decompressive hemicraniectomy was performed.

### Definition of admission parameters

Standard demographic data were acquired from hospital charts, including age and sex. Time of trauma was defined as when the alarm came in to the regional alarm central and was acquired through pre-hospital charts. Glasgow Coma Scale (GCS) at admission to the hospital was used and handled as a continuous, ordinal scale in the analyses as previously suggested.^33^ Pupil reaction to light was assessed at hospital admission and defined as either both reactive, unilaterally unresponsive, or bilaterally unresponsive. Pre-hospital hypoxia was defined as either an oxygen saturation <90%, or if saturation was not available if the airway was deemed obstructed at the scene of accident. Pre-hospital hypotension was defined as a systolic blood pressure <90 mm Hg. Glucose and hemoglobin sampled at admission to the hospital were acquired. These variables create the foundation, together with CT parameters, of the IMPACT study group's TBI outcome prediction calculator.^3^

Abbreviated Injury Scale (AIS), Injury Severity Score (ISS), and New Injury Severity Score (NISS) were assessed by ISS-trained specialist nurses.^34,35^ Significant extracranial multi-trauma, as per previous definitions,^33^ was noted.

### Neuroradiology

CT scans at admission were assessed using Marshall CT classification, as well as Rotterdam and Stockholm CT score.^36–38^ Because the admission CT was used, all patients with focal mass lesions (>25 cm^3^) were considered Marshall VI. Any progression of intracranial hemorrhages between the first and second CT scans was noted.

For a subset of patients where the CT scan and/or clinical course suggested presence of diffuse axonal injury (DAI), magnetic resonance imaging (MRI) was performed as per clinical routine. The clinical MRI protocol consisted of echo planar diffusion–, fluid attenuated inversion recovery (FLAIR)-, gradient echo (GRE)-, and T_1_- and T_2_-weighted image sequences. Either hemorrhagic and/or non-hemorrhagic DAI was noted.

### Clinical outcome

Long-term functional outcome was assessed using the five-stage Glasgow Outcome Score (GOS), where 1 = death, 2 = persistent vegetative state, 3 = severe disability (dependent state), 4 = moderate disability (independent state), and 5 = recovery (including low-grade disability).^39^ The GOS was assessed prospectively through a quality-of-life questionnaire, including questions from structured GOS interviews, that was sent to the patient at 12 months after injury, or in case these were not returned, it was recorded at visits to the outpatient clinic. The date for GOS assessment was noted.

### Blood sampling

Blood was pragmatically sampled, depending on research staff availability, for NF-L, GFAP, UCH-L1, and tau analyses. These were sampled up to three times during the first 2 weeks, if the patient did not die or was discharged. Serum blood collection tubes where used (yellow cap, clot activator with gel). Sampling was performed by arterial lines and then transported to a local biobank where they stood upright for 30 min to allow coagulation. Samples where then centrifuged for 15 min at 2000g and aliquoted and stored at −80°C.

Blood for analysis of S100B and NSE were drawn twice-daily and sent directly to the Department of Clinical Chemistry at the Karolinska University Hospital, as per clinical routine.

### Serum analysis

Serum GFAP, UCH-L1, NF-L and tau concentrations were measured using the Human Neurology 4-Plex A assay (N4PA) on an HD-1 Single molecule array (Simoa) instrument, according to instructions from the manufacturer (Quanterix, Lexington, MA). Measurements were performed by board-certified laboratory technicians, who were blinded to clinical data, in one round of experiments using one batch of reagents and with baseline and follow-up samples analyzed side by side. In the assay, calibrators were run as duplicates, samples as singlicates, and two internal quality-control plasma samples were run in the beginning and the end of each run. Between-day coefficient of variations (CVs) were 8.8% at 103 pg/mL and 8.4% at 7.4 pg/mL for NF-L, 11.9% at 1.2 pg/mL and 9.7% at 22.5 pg/mL for tau, 9.0% at 72 pg/mL and 7.3% at 88 pg/mL for GFAP, and 33.3% at 11.4 pg/mL and 34.0% at 10.8 pg/mL for UCH-L1.

All serum S100B samples collected until September 2008 were analyzed at Karolinska University Hospital, Department of Clinical Chemistry, using a quantitative automated luminometric immunoassay (LIAISON-mat S100 system; DiaSorin, Sangtec, Italy). In September 2008, the Department changed to an automatic electrochemiluminescence immunoassay (Elecsys S100B; Roche Diagnostics, Penzberg, Germany). A good correspondence between the two methods has been shown, including internal validation by the Department of Clinical Chemistry, Karolinska University Hospital, Solna, Sweden.^6^ Serum NSE samples were analyzed using an immunoradiometric assay (Liaison; DiaSorin) throughout the whole study at the Karolinska University Hospital, Department of Clinical Chemistry. The detection levels for the LIAISON ranges from 0.04 μg/L for NSE and 0.02 μg/L for S100B, whereas the Elecsys detects serum S100B levels down to 0.005 μg/L. No patient presented with concentrations below detection levels.

### Statistical analysis

Data are presented as median and interquartile range (IQR) for continuous data and grouped for categorical data. Univariate logistic regressions toward different levels of the GOS (proportional odds analysis, using “rms” package in R) were applied to study the predictive ability of admission/IMPACT parameters as well as the protein biomarkers. Similar tests were performed toward different CT severity scores for the biomarkers, as well as linear models, were applicable. Protein biomarkers were also tested versus different dichotomizations of outcome, GOS1–3 (unfavorable) versus GOS4–5 (favorable), and GOS1 (dead) versus GOS2–5 (alive).

Nagelkerke's pseudo-*R*^2^ was used to determine model performance and was bias adjusted for multiple parameters using a bootstrap method. Nagelkerke's pseudo-*R*^2^ describes performance as between “0” and “1,” where 1 fully explains the model, akin to explained variance. AUC (area under the curve) ROC (receiver operating characteristics) curves were also used to assess model performances (using the pROC package in R). Step-up, as well as a step-down, multi-variable models were used to examine how the biomarkers added explained variability to the baseline outcome predictors used in the IMPACT calculator.^3^ This was done for unfavorable versus favorable outcome as per IMPACT. A sliding window, assessing a proportional odds analysis of the biomarkers toward the GOS and with bootstrapped confidence intervals, was used to assess the prediction performance of a biomarker over time, after trauma.

The R package “ggplot2” and conditional density plots were used to illustrate biomarker data.^40^ Linear correlations (Spearman) were used to determine cross-correlations between the different protein biomarkers, using peak concentrations so that only 1 patient contributed one time point (visualized with “GGally” package). Principal component analysis (PCA) of the biomarkers was performed using R (“prcomp” package) to explore potential protein interactions.^41^ A loading plot, highlighting potential covariance among the biomarkers in the two first components, was created. The statistical program R was used in the analyses with the interface R-studio (Boston, MA).^42^ A *p* value of <0.05 was considered significant.

### Missing data

All univariate regression models performed were done with unimputed data, excluding missing observations. The multi-variable prediction models were performed after multiple imputation (MI; “MICE” package, R), as advocated in the statistical literature and by the IMPACT TBI study group.^43,44^ We applied seven imputed sets of data in our MI, with the imputed data coming from a regression imputation with each imputation drawn from a distribution, retaining uncertainty caused by imputation in analyses toward the dichotomized outcomes. All seven models were checked for consistency, and an average Nagelkerke's pseudo-*R*^2^ of these seven imputations was reported. Patients that had no corresponding S100B/NSE analyses for the three sampling time points in the multi-variable models had their peak concentrations of S100B and NSE imputed.

## Results

### Patient demographics

A total of 502 NCCU TBI patients were admitted between 2007 and 2013, and of these, 172 had been included in the study. Patients were predominantly male (76%) with a median age of 55 years (Table 1). Most patients were unconscious upon admission to the hospital (GCS3–8; 70%). As per trauma definitions, 28% had significant extracranial injury and the cohort had a relatively high overall trauma severity, with 86% of patients having either an AIS of 4 (“severe”) or 5 (“critical”), with median ISS of 25 and median NISS of 43, indicating that they had more-severe brain injuries than extracranial injuries (Supplementary Table S1). The 1-year mortality was 13%, and the rate of unfavorable outcome (GOS1–3) was 50% (Table 1).

**Table 1.** Patient Demographics

*Admission characteristics*	*Subcategory/units*	n* = 172 patients*
Sex	Male/female (%/%)	130/42 (76/24)
Age	Median (IQR)	55 (38–62)
Scene of accident hypoxia	Yes	29 (17%)
	Missing data	8 (5%)
Scene of accident hypotension	Yes	3 (2%)
	Missing data	43 (25%)
Admission GCS	3–8	121 (70%)
	9–13	38 (22%)
	14–15	13 (8%)
Admission pupil responsiveness	Normal	125 (73%)
	Unilateral unresponsiveness	18 (10%)
	Bilateral unresponsiveness	24 (14%)
	Missing data	5 (3%)
Admission hemoglobin	g/dL, median (IQR)	136 (123–146)
	Missing data	7 (4%)
Admission glucose	mmol/L, median (IQR)	7.9 (7.0–9.8)
	Missing data	30 (17%)
Functional outcome		
GOS1 (death)	*n* (%)	21 (12%)
GOS2 (vegetative state)	*n* (%)	0
GOS3 (severe disability, dependent state)	*n* (%)	63 (37%)
GOS4 (moderate disability, independent state)	*n* (%)	49 (28%)
GOS5 (mild or no disability)	*n* (%)	39 (23%)
Time from trauma to GOS assessment (living patients)	Days, median (IQR)	366 (343–383)

Patient demographics for the included patients.

GCS, Glasgow Coma Scale; GOS, Glasgow Outcome Scale; IQR, interquartile range.

### Sampling

**FIG. 1.**
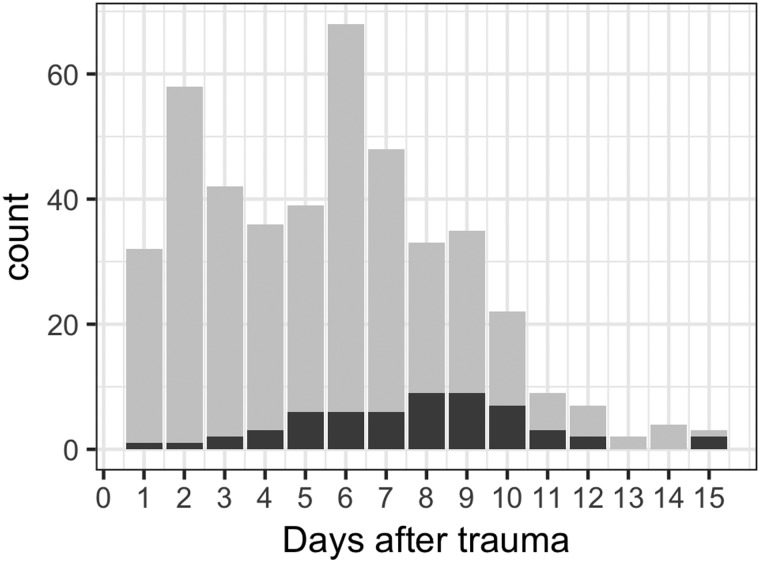
Sample counts days post-trauma. Counts of samples by day post-trauma (gray, n = 421; x-axis, days; y-axis, number of samples [counts]) and counts where exact corresponding times of NSE and S100B did not exist (black, *n* = 61). NSE, neuron-specific enolase; S100B, S100 calcium-binding protein B.

In total, 421 samples of GFAP, UCH-L1, tau, and NF-L were available, acquired at median 3, 6, and 9 days after trauma (Fig. 1; Supplementary Table S2). All 172 patients had at least one sample, *n* = 146 (85%) had two samples, and *n* = 95 (55%) had three samples that could be analyzed. These corresponded with *n* = 363 S100B and *n* = 360 NSE samples, totaling 2407 biomarker measurements. We primarily choose the peak concentration per patient in our analyses because we believe it to contain the most biologically relevant information, which was, except for NF-L (20%), almost exclusively the first sample (73–95%; Supplementary Table S2).

### Univariate correlation versus injury severity

The CT scoring systems were used as surrogate markers for injury severity. In general, S100B and UCH-L1 had the strongest correlations with Stockholm CT scores and exhibited the greatest Nagelkerke's pseudo-*R*^2^ in models predicting Rotterdam CT scores, as well as to hemorrhagic progression between the first and second CT scan (Table 2; Supplementary Fig. S1A–C). Interestingly, in models predicting extracranial trauma, the first sample acquired for S100B and UCH-L1 displayed significant associations (Table 2), whereas no significance was found for the first sample for the other biomarkers. The other biomarkers were associated, albeit less, with increasing severity on the CT scoring systems. Marshall CT classification had the weakest associations, but a higher Marshall (focal injury) was still significant for all biomarkers except tau and NF-L.

**Table 2.** Associations between Protein Biomarkers and Injury Severity

	*S100B*	*NSE*	*GFAP*	*UCH-L1*	*Tau*	*NF-L*
CT parameters (first sample)						
Marshall CT classification (*p* value, adjusted *R*^2^)	0.002 (0.058)	0.040 (0.027)	0.004 (0.050)	0.015 (0.037)	0.072 (NS)	0.982 (NS)
Rotterdam CT score (*p* value, adjusted *R*^2^)	<0.001 (0.077)	0.005 (0.043)	0.002 (0.047)	<0.001 (0.092)	<0.001 (0.082)	0.078 (NS)
Stockholm CT score (*p* value, correlation coefficient)	<0.001 (0.298)	0.004 (0.210)	0.011 (0.179)	<0.001 (0.283)	<0.001 (0.255)	0.024 (0.155)
Hemorrhagic progression between first and second CT (*p* value, adjusted *R*^2^)	<0.001 (0.099)	<0.001 (0.095)	0.009 (0.032)	<0.001 (0.116)	<0.001 (0.081)	0.003 (0.047)
MRI subgroup *n* = 42 (diffuse injury on CT, peak concentration)						
DAI (yes/no)	0.059 (negative correlation)	0.104 (negative correlation)	0.471 (negative correlation)	0.104 (negative correlation)	0.043 (negative correlation)	0.145 (positive correlation)
Extracranial injury (first sample)						
Multi-trauma (*p* value, adjusted *R*^2^)	0.002 (0.091)	0.169 (NS)	0.079 (NS)	0.006 (0.056)	0.298 (NS)	0.861 (NS)

Associations between protein biomarkers and intracranial/extracranial injury. Logistic or linear regression models, where appropriate, were used to performed the analyses. Nagelkerke's pseudo-*R*^2^ is described if statistically significant (*p* < 0.05). For Stockholm CT scores, linear correlation models were used and correlation coefficients presented.

CT, computerized tomography; DAI, diffuse axonal injury; GFAP, glial fibrillary acidic protein; MRI, magnetic resonance imaging; NF-L, neurofilament-light; NSE, neuron-specific enolase; NS, non-significant; S100B, S100 calcium-binding protein B; UCH-L1, ubiquitin carboxyl-terminal hydrolase-L1.

A total of 81 patients had an MRI performed because of suspected DAI injuries, and 41 of these also had diffuse injury (Marshall classification I–IV) on their admission CT scan. In this subgroup, no biomarker was significantly increased in DAI (*n* = 29) versus non-DAI (*n* = 12) patients (Table 2).

### Univariate correlations versus outcome

Among the known IMPACT predictors, only age and glucose levels were significantly correlated to outcome in the logistical regression model (Table 3). Other strong associations in the collected clinical data included progression of hematoma between the first and second CT scan (pseudo-*R*^2^, 0.158), as well as the Stockholm CT score on the initial scan (pseudo-*R*^2^, 0.226; Table 3).

**Table 3.** Univariate Analyses versus Patient Outcome

*Parameters*	p *value*	*Nagelkerke's pseudo-*R^*2*^	*AUC (95% CI)*
Admission			
Age	<0.001	0.191	0.733 (0.658–0.808)
GCS at admission	0.106	NS	0.569 (0.484–0.654)
GCS at scene of accident	0.014	0.047	0.608 (0.524–0.699)
Pupil responsiveness at admission (as factor)	0.120	NS	0.568 (0.501–0.634)
Hemoglobin levels at admission	0.061	NS	0.578 (0.490–0.665)
Glucose levels at admission	0.015	0.054	0.593 (0.499–0.688)
Scene of accident hypoxia	0.131	NS	0.545 (0.486–0.603)
Scene of accident hypotension	0.565	NS	0.492 (0.466–0.519)
CT scan			
Marshall CT classification	0.622	NS	0.512 (0.440–0.595)
Rotterdam CT score	0.033	0.035	0.584 (0.502–0.666)
Stockholm CT score	<0.001	0.226	0.742 (0.669–0.816)
Progression of hemorrhage	<0.001	0.158	0.656 (0.592–0.721)
Trauma scores			
Head-AIS	0.044	0.032	0.589 (0.513–0.666)
NISS	0.019	0.044	0.605 (0.517–0.693)
ISS	0.110	NS	0.562 (0.475–0.649)
Significant multi-trauma	0.514	NS	0.476 (0.408–0.544)
Biomarkers GOS1–3 vs 4–5 (unfavorable vs. favorable)			
S100B peak concentration	<0.001	0.213	0.708 (0.630–0.787)
NSE peak concentration	<0.001	0.085	0.604 (0.518–0.690)
GFAP peak concentration	<0.001	0.217	0.724 (0.648–0.800)
UCH-L1 peak concentration	<0.001	0.211	0.742 (0.670–0.815)
Tau peak concentration	<0.001	0.162	0.708 (0.631–0.786)
NF-L peak concentration	<0.001	0.154	0.699 (0.622–0.776)
Biomarkers GOS 1 versus 3 versus 4 versus 5 (proportional odds)			
S100B peak concentration	<0.001	0.197	0.729
NSE peak concentration	<0.001	0.096	0.609
GFAP peak concentration	<0.001	0.174	0.741
UCH-L1 peak concentration	<0.001	0.271	0.749
Tau peak concentration	<0.001	0.207	0.713
NF-L peak concentration	<0.001	0.101	0.639
Biomarkers GOS 1 versus 2–5 (dead vs. alive)			
S100B peak concentration	<0.001	0.218	0.822 (0.720–0.923)
NSE peak concentration	<0.001	0.132	0.645 (0.480–0.810)
GFAP peak concentration	<0.001	0.203	0.814 (0.698–0.930)
UCH-L1 peak concentration	<0.001	0.342	0.828 (0.722–0.933)
Tau peak concentration	<0.001	0.273	0.787 (0.680–0.895)
NF-L peak concentration	0.041	0.046	0.651 (0.534–0.767)

Univariate logistic regression displaying Nagelkerke's pseudo-*R*^2^ and AUC of admission parameters and biomarker levels versus long-term patient outcome in different dichotomizations. “Admission,” “CT scan,” and “Trauma score” parameters used the GOS1–3 versus 4–5 (unfavorable vs. favorable) outcome dichotomization. Nagelkerke's pseudo-*R*^2^ is described if statistically significant (*p* < 0.05). For multi-level receiver operating characteristics (ROC) calculations, only AUC can be presented.

AUC, area under curve; CI, confidence interval; GCS, Glasgow Coma Scale; CT, computerized tomography; AIS, Abbreviated Injury Score; NISS, New Injury Severity Score; ISS, Injury Severity Score; GOS, Glasgow Outcome Scale; NS, non-significant; GFAP, glial fibrillary acidic protein; NF-L, neurofilament-light; NSE, neuron-specific enolase; S100B, S100 calcium-binding protein B; UCH-L1, ubiquitin carboxyl-terminal hydrolase-L1.

In the univariate models predicting levels of GOS and mortality, UCH-L1 explained the most variation (pseudo-*R*^2^, 0.271 and 0.342, respectively). GFAP was slightly better than the other markers in predicting the favorable versus unfavorable GOS outcome dichotomization (pseudo-*R*^2^, 0.217; S100B pseudo-*R*^2^, 0.213; Table 3). However, in general, S100B, UCH-L1, tau, and GFAP performed similarly between the outcome representations, whereas peak NSE and NF-L levels performed worse (Table 3; Supplementary Fig. S2).

### Protein biomarkers versus outcome over time

**FIG. 2.**
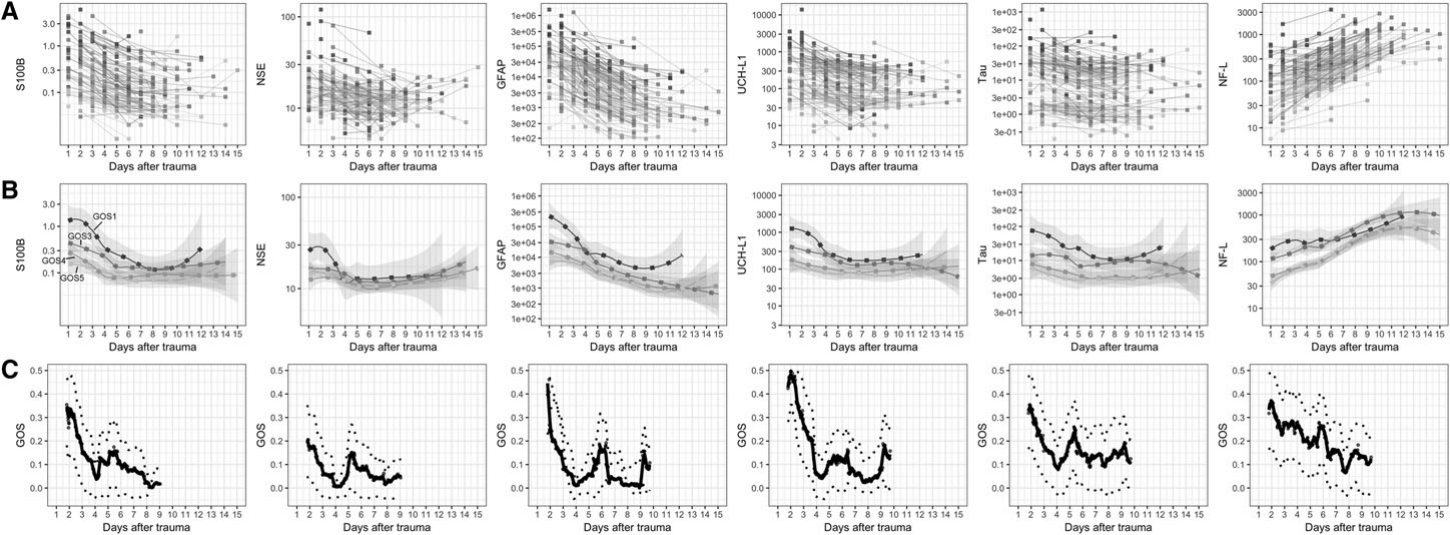
Biomarker dynamics over time stratified by outcome level. (**A**) Biomarker trajectories of all individuals gray graded by outcome (darker = lower GOS). The y-axis units for S100B (log, μg/L), NSE (log, μg/L), GFAP (log, pg/mL), UCH-L1 (log, pg/mL), Tau (log, pg/mL), and NF-L (log, pg/mL). (**B**) GOS grouped biomarker levels (group mean) over time (darker = lower GOS). The y-axis as in (A). The shown shaded 95% confidence levels are seen to widen as data become sparse. (**C**) Strength of association of biomarker levels toward outcome (GOS) over time (days). Univariate outcome prediction accuracy using proportional odds (Nagelkerke's pseudo-*R*^2^, y-axis) of unlogged biomarker data, within a sliding window of 200 data points is shown with a LOWESS curve fit and bootstrapped confidence interval (2 SDs). The mean of the time points in the sliding window is used, thus presenting no prediction values day 1. GFAP, glial fibrillary acidic protein; GOS, Glasgow Outcome Scale; LOWESS, LOcally WEighted Scatter-plot Smoother; NF-L, neurofilament-light; NSE, neuron-specific enolase; S100B, S100 calcium-binding protein B; SD, standard deviation; UCH-L1, ubiquitin carboxyl-terminal hydrolase-L1.

The outcome predictions as observed in the univariate regressions (Table 3) can be better elucidated by visualizing protein concentrations over time in relation to the trauma with stratification according to different levels of GOS (Fig. 2). In general, S100B, NSE, GFAP, UCH-L1, and tau presented with initially high levels that decreased over time, whereas NF-L generally increased over time during the study period (Fig. 2). All biomarkers displaying a good discriminatory capacity in the regression models had distinctly higher levels in the group of patients with unfavorable outcome (Fig. 2A). The best predictive period for all markers, except NF-L, could be observed within 4–5 days of trauma (Fig. 2B), where the pseudo-*R*^2^ favorable/unfavorable outcome is approximately 0.300–0.400 for most proteins. A similar result was noted for mortality and different stages of GOS over time after trauma (Supplementary Table S3; Supplementary Fig. S3).

### Multi-variable analysis versus outcome

By combining the IMPACT outcome predictors age, admission GCS and pupil responsiveness, pre-hospital hypoxia and hypotension, as well as admission hemoglobin and glucose levels together with the Rotterdam CT score, a Nagelkerke's pseudo-*R*^2^ of 0.285 was reached in predicting favorable/unfavorable outcome (Table 4).^3^ We found a substantial increase in the prediction model if the Stockholm CT score was used instead of Rotterdam for CT injury characteristics, so this model (with a pseudo-*R*^2^ of 0.375) was used as our “Base” model.

**Table 4.** Multi-Variable Analyses versus Patient Outcome

*Unfavorable versus favorable outcome*	*Nagelkerke's pseudo-*R^*2*^
IMPACT Rotterdam model	0.285
Base model (IMPACT but Stockholm CT instead of Rotterdam CT)	0.375
Base + S100B	0.463^a^ (*p* = 0.003)
Base + NSE	0.406
Base + UCH-L1	0.458
Base + Tau	0.445
**Base + GFAP**	**0.470^a^ (*p* = 0.017)**
Base + NF-L	0.450^a^ (*p* < 0.001)
Base + GFAP + S100B	0.487
Base + GFAP + NSE	0.470
Base + GFAP + UCH-L1	0.475
Base + GFAP + Tau	0.479
**Base + GFAP + NF-L**	**0.514^b^ (*p* = 0.001)**
**Base + GFAP + NF-L + S100B**	**0.522** (*p* = 0.223)
Base + GFAP + NF-L + NSE	0.514
Base + GFAP + NF-L + UCH-L1	0.515
Base + GFAP + NF-L + Tau	0.514

Multi-variable regression analyses versus unfavorable/favorable (GOS1–3 vs. 4–5) outcome at 12 months. The IMPACT model consists of age, GCS, pupil response, scene of accident hypoxia, scene of accident hypotension, admission glucose, and admission hemoglobin. To this, Rotterdam CT score was added initially, but then replaced by Stockholm CT-score forming the “Base” model used. The model exhibiting highest pseudo-*R*^2^ is highlighted in bold. Significantly better models according to the likelihood ratio test are shown with *p* values, stepping up from the nested base model (Base, Base + GFAP or Base + GFAP + NF-L).

^a^ Step-up model significantly improved compared to the Base model.

^b^ Step-up model significantly improved compared to Base + GFAP model. *p* value for Base + GFAP + NF-L + S100B highlighted to show that it did not yield independent information over the Base + GFAP + NF-L model.

IMPACT, International Mission for Prognosis and Analysis of Clinical Trials in TBI; CT, computerized tomography; GFAP, glial fibrillary acidic protein; NF-L, neurofilament-light; NSE, neuron-specific enolase; S100B, S100 calcium-binding protein B; UCH-L1, ubiquitin carboxyl-terminal hydrolase-L1.

#### Step-up model

Each individual biomarker added significant, independent information to the base model, but only S100B, GFAP, and NF-L significantly. NSE added least, whereas the others added in the vicinity of an additional 0.08–0.09 pseudo-*R*^2^ (Table 4). GFAP added marginally more than the others and was therefore used as base for the next step-up. The Base + GFAP model exhibited a pseudo-*R*^2^ of 0.470, and adding the other markers revealed that NF-L added the most additional variability to that model, now totaling a pseudo-*R*^2^ of 0.514. Adding S100B to that dual-marker model resulted in an additional increase to 0.522 (though a non-significantly better model, *p* = 0.223), which was also the maximum pseudo-*R*^2^ reached, including all biomarkers.

Additionally, in an exploratory approach, we also tested how well the biomarkers did in a proportional odds model using GOS as an ordinal scale. Here, similarly to what was observed in the univariate analyses, the best model contained UCH-L1 and additional independent information was provided by NF-L (Supplementary Table S4).

#### Step-down model

The step-down model contained all Base variables grouped together as one, as well as the peak concentrations of all the biomarkers. The model left the Base variable together with peak levels of GFAP and NF-L.

In predicting different levels of GOS utilizing a proportional odds model, apart from the Base variable, UCH-L1 remained as the only significant biomarker.

### Covariance between the biomarkers

A cross-correlation matrix revealed that some of the peak serum concentration of the biomarkers had a very strong correlation, especially GFAP, UCH-L1, and tau (correlation-coefficients, 0.83–0.88; Table 5). S100B, NSE, and NF-L exhibited significant, but lower, cross-correlations, approximately 0.50–0.25, with NF-L showing the lowest relations to the other markers (Supplementary Fig. S4).

**Table 5.** Cross-Correlation Analyses between Different Protein Biomarkers

*Peak serum levels*						
	*S100B*	*NSE*	*GFAP*	*UCH-L1*	*Tau*	*NF-L*
S100B	1.000					
NSE	0.458	1.000				
GFAP	0.670	0.496	1.000			
UCH-L1	0.665	0.486	0.880	1.000		
Tau	0.632	0.548	0.824	0.877	1.000	
NF-L	0.279	0.282	0.297	0.383	0.438	1.000

Cross-correlation analyses displaying Spearman's rho correlation coefficient for peak serum levels for each patient. This was done in patients where all biomarker levels where present, thus *n* = 168.

GFAP, glial fibrillary acidic protein; NF-L, neurofilament-light; NSE, neuron-specific enolase; S100B, S100 calcium-binding protein B; UCH-L1, ubiquitin carboxyl-terminal hydrolase-L1.

**FIG. 3.**
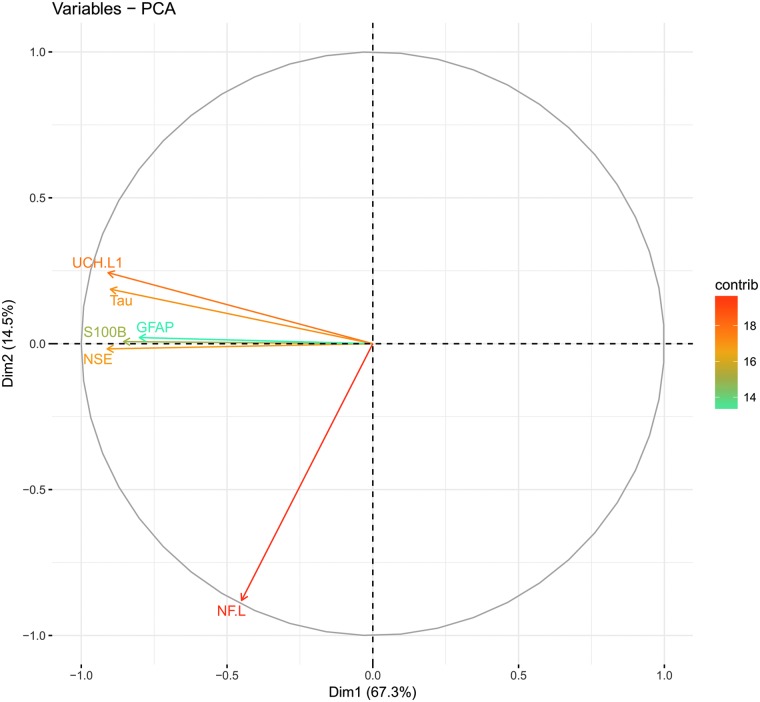
Principal component analysis of biomarkers. A principal component analysis (PCA) of the first two dimensions of the biomarker data explaining 81.8% of the data variance. Dimension1 (Dim1, x-axis) explains 67.3% of the variance and Dim2 (y-axis) an additional 14.5%. The heatmap indicates how well each biomarker is explained (%) by these two components (vector length). Biomarkers can be seen to have substantial covariance except in the case of NF-L, suggesting it to contain highly different information. GFAP, glial fibrillary acidic protein; NF-L, neurofilament-light; NSE, neuron-specific enolase; S100B, S100 calcium-binding protein B; UCH-L1, ubiquitin carboxyl-terminal hydrolase-L1. Color image is available online.

The PCA analysis revealed that the first two components explained around 82% of the variance of the data (Supplementary Table S5), primarily in the first component, with subsequent components explaining substantially less. The predominantly neuronal proteins (except NSE) UCH-L1 and tau clustered tightly (Fig. 3). The predominantly astrocytic proteins S100B and GFAP clustered together with NSE, whereas the axonal NF-L showed a separate trajectory (Fig. 3).

## Discussion

We here combined six of the most commonly used TBI biomarkers in an extensive prospective cohort of NCCU TBI patients and found that biomarker levels do, in comparison to other known outcome predictors in TBI, explain more variability in different outcome prediction models. All biomarkers except NSE provided significant information toward all dichotomizations of outcome in univariate analyses, with GFAP and UCH-L1 being the strongest predictors.

Adjusting for IMPACT variables in a multi-variable analysis, combining GFAP and NF-L provided the best enhancement of performance in predictive models, resulting in a pseudo-*R*^2^ increase from 0.375 to an impressive 0.522. Compared to the other markers, the strongest covariances were found between GFAP, UCH-L1, and tau, with correlation coefficients reaching 0.83–0.88, in turn explaining why these markers presumably did not add independent information in combination. Further, the PCA model revealed clustering of proteins with similar cellular origin and/or temporal profiles, grouping the predominantly neuronal markers tau and UCH-L1 and the astrocytic markers S100B and GFAP as well as the temporally different axonal protein NF-L. Collectively, these findings highlight the strong predictive capabilities of serum protein biomarkers in TBI outcome models and that combination of different markers enhances the precision further.

All markers were associated with severity of TBI, as assessed on admission CT scans, however with different degrees of association depending on type of CT scoring. The best association was found to be with the Stockholm CT score, which includes grading of traumatic subarachnoid hemorrhage specifically.^38^ In contrast, the Rotterdam CT score is more focused on mass-effect components and, perhaps more appropriate, as a marker of affected parenchymal volume,^37^ whereas the Marshall CT classification mainly discriminates between diffuse and focal injuries.^36^ None of the markers were significantly associated with DAI detected on MRI. The analyses reached only borderline significance and being *lower* in patients with DAI (tau *p* = 0.043 and S100B *p* = 0.059), presumably because the non-DAI patients still had had more mixed-density lesions with greater parenchymal volume affected, though smaller than 25 cm^3^. The sensitivity for detecting a true difference may be greater if patients with only DAI are selected as done by Ljungqvist and colleagues, where they studied TBI patients with low concentrations of S100B and found that, in this small (*n* = 9) cohort, NF-L levels were associated with extent of DAI injuries on diffusion tensor MRI.^45^

Although being correlated with both TBI severity on admission CT scans and relevant outcomes, early S100B and UCH-L1 levels were also associated with presence of associated non-cranial injuries, replicating previous findings.^46–49^ The other biomarkers displayed limited correlations with extracranial injury, in line with their restricted expression to nervous tissues.^27^ Notably, the temporal sliding window approach used in this study and the relatively few samples in the first 24 h likely will underestimate the impact of extracranial contribution (Supplementary Fig. S3C). In fact, in a previous study, we found a strong influence of extracranial injuries on S100B and NSE in the first 72 h with more-frequent sampling in the earlier phases,^6^ when extracranial contribution likely is most important.

All biomarkers used in the study have been previously correlated to outcome in a variety of outcome models.^15,28,31,50–64^ However, unknown timing of injury, measurement of single or a few markers, use of different outcome dichotomizations, and different statistical methods make it practically impossible to say whether one biomarker is superior to another. It is therefore noteworthy that we found individual biomarkers to be better outcome predictors than many other known single predictors, including age, GCS, pupil responsiveness, and most CT scoring systems. Looking specifically at the biomarkers, UCH-L1 performed particularly well against all different outcome dichotomizations used, closely followed by GFAP, S100B, and tau. In contrast, the predictive values of NF-L and NSE were lower, especially NSE, which could only predict mortality, also in line with work from us and others.^6,28^ It should be noted that NSE levels may be affected by hemolysis of samples,^65^ presumably common in our clinical scenarios. Among the included markers, UCH-L1 has the shortest effective serum half-life (7–10 h in severe TBI),^62^ which could explain its higher performance, given that a more-severe brain injury will yield a prolonged release.

By visualizing temporal profiles in relation to GOS levels, interesting features of the biomarkers emerge. All biomarkers, except for NF-L, exhibited a decrease in concentration over the first days after injury, leveling at approximately 4 days after trauma. A clear separation of patients that died (GOS1), or had unfavorable outcome (GOS 1–3), can be seen during this time window, indicative of a continued cerebral efflux presumably attributed to ongoing cell death.^9^ This is in line with several previous studies looking at S100B, GFAP, NSE, and UCH-L1,^31,59,66,67^ whereas it has not been reported yet for tau. NF-L clearly stands out among the included markers, given that levels kept increasing throughout the study period, in accord with previous work.^15^

Although previous reports found that UCH-L1 (10 h), tau (10 h), and S100B (24 h) have shorter effective half-lives in serum as compared to GFAP (48 h), NSE (48h), and NF-L (>1 weeks) in patients with severe TBI,^25,26^ this is not entirely evident in the current data set. However, it does appear that it takes longer for GFAP to level out as compared to S100B, UCH-L1, and NSE. This is probably also attributed to the low sampling frequency in this study and the subsequently lower resolution of information than in our previous study for S100B and NSE, which observed rather steep declines after 24 h.^6^ In the present study, NF-L increased over the first 2 weeks, which may relate to a longer effective half-life in serum in addition to release from degeneration of axonal connections with a longer time frame than more-acute necrotic cell death in the impact core.^12^ Thus, NF-L may be more suitable for sampling in more chronic stages of TBI (weeks to months after trauma) and could explain its relatively low predictive power, if used individually, in this study.

More information on the mechanism of efflux of these biomarkers from the cerebral compartment in the context of TBI is also warranted.^68^ It has been speculated that blood–brain barrier (BBB) disruption,^69^ glymphatic system activity independent of BBB disruption,^70^ or a more-passive release from brain/cerebrospinal fluid^71^ may contribute to efflux. Clearance from serum is also not well described, where only S100B has been studied and has shown a renal clearance.^72^ Other larger proteins are believed to be, at least in part, deaminated and metabolized by the liver, thus also potentially affecting serum levels.^73^ Moreover, they could also be proteolytically processed into smaller fragments no longer recognized by the assays.

GFAP and NF-L added the most independent information when predicting unfavorable outcome in multi-variable models, increasing pseudo-*R*^2^ from 0.38 to 0.51. An additional step up with other biomarkers did not significantly improve the models. In theory, adding proteins with different cellular origins or kinetic profiles could contain different information regarding pathophysiological processes, and might be expected to represent the best combination in outcome models.

We have previously noted, for NF-L and S100B, that both markers in combination performed better than either used alone, and that addition of NSE did not provide additional predictive information.^6,29^ Czeiter and colleagues noted, in a study of 45 patients with severe TBI using a more limited IMPACT model (a “core” model consisting of only age, GCS motor score, and pupil abnormalities), that GFAP added 0.162 pseudo-*R*^2^ to this model,^30^ which is, to some extent, similar to our study, but also demonstrates that the other IMPACT and CT parameters retain a lot of the predictive information in these models. Gradisek and colleagues reported similar findings, that is, that S100B and GFAP, but not NSE, added independent information in outcome prediction models using some admission parameters and CT characteristics predicting mortality,^56^ as was also replicated by Vos and colleagues, where S100B and GFAP performed better in outcome prediction models than if GFAP and NSE were used in the presence of admission parameters.^28^

GFAP, UCH-L1, and tau exhibited strong intercorrelations, with correlations coefficients ranging from 0.83 to 0.88. Tau levels have not been extensively correlated to other biomarker concentrations, but GFAP and UCH-L1 correlation coefficients have been shown to be 0.24 to approximately 0.50,^74–79^ and thus substantially less than in our study. However, these studies mainly included patients with mild TBI, many without intracranial lesions. The study by Korley and colleagues noted in mild TBI that GFAP was slightly better than UCH-L1, NF-L, and tau, but that a model detecting CT positive scans increased from an AUC of 0.88 using GFAP to 0.90 using all the biomarkers together, so a substantial covariance is likely present between markers.^79^ In comparison, TBI patients in need of NCCU care and intracranial monitoring usually suffer from a mix of intra- and extracranial injuries, where biomarker serum release patterns are probably different.

The coefficients observed between S100B, NSE, and other markers in this study are similar to what have been described previously.^28,67,74,80^ NF-L levels did not correlate well with the other markers, especially S100B and NSE, likely because of a difference in the underlying pathophysiology for its release, as well as a potentially different clearance pattern. The PCA revealed a distinct clustering of the primarily neurological markers, tau and UCH-L1, that exhibited similar projections in the first two components. The primarily astrocytic markers, S100B and GFAP, also clustered together with the neuronal NSE. In contrast, NF-L with an axonal origin displayed the most unique projection among the markers analyzed here. These findings are in concordance with the multi-variable outcome models and the information content provided by the different biomarkers. The different components probably also indicate different temporal trajectories for the proteins, explaining the very different clustering for NF-L, and why tau and UCH-L1 clustered so closely, as they share temporal patterns.

These findings are difficult to relate to previous work, given that few studies have used this approach before. However, Mondello and colleagues reported on a tentative “glial:neuronal ratio” by creating a ratio between serum levels of GFAP and UCH-L1,^81^ suggesting that patients with more-focal mass lesions on admission CT scans displayed higher GFAP levels, whereas more-diffuse injuries instead released predominantly UCH-L1. In our study, these two markers positioned themselves differently, especially based on the second component, supporting that they might be markers for different underlying pathophysiology (even if not significantly associated with either diffuse or focal injury using Marshall CT, *p* = 0.359 data not shown).

In an exploratory analysis, we tested how interactions between biomarkers improved outcome prediction and the only significant biomarkers, if used in a similar ratio, was S100B:GFAP, adding information if S100B and GFAP independently were used in the same outcome prediction model (data not shown). This was presumably attributed to the difference in temporal profiles more than cellular origin.^25^ GFAP was also the protein with least explained variance by the two first principal components, suggesting that it carries different biological information. In summary, the PCA indicated distinct clusters of proteins, supporting that biomarkers of separate cellular origins and temporal profiles may contribute differently in prediction models.

### Limitations

Patients were sampled once to thrice over the first 2 weeks, where more-frequent sampling would have provided more-precise information on temporal profiles. Moreover, the timing of the first sample in relation to injury varied considerably between patients, which likely affected outcome prediction modeling negatively, given that peak values may have been missed. This is specifically limiting for proteins with a shorter effective serum half-life as compared to NF-L.

In cases of milder TBI, GFAP concentrations have been shown to peak at around 24 h, in comparison to UCH-L1, which has a steadier decline.^82^ Presumably, most pathophysiological information is acquired from these “peak” levels, which we have observed in our studies with S100B.^11^ In fact, in an exploratory approach, if the peak levels 12–36 h after trauma of S100B were used, it outperformed GFAP in the multi-variable outcome prediction models (data not shown), stressing the need to find the ideal time point for each protein. However, the relatively large patient population with non-set-sampling time points may, to some extent, offset these limitations, though the findings presented herein should be replicated by studies with a more-structured sampling procedure, such as CENTER-TBI (Collaborative European NeuroTrauma Effectiveness Research in Traumatic Brain Injury),^83^ which would also allow for external validation our suggested models.

Patients were pragmatically included, depending on the availability of researchers and staff. Thus, though not all NCCU TBI patients were included, we believe the material represents a valid cohort, with more severely injured patients recruited, which is our target population, and that the data are not affected by selection bias.

The lack of a diffuse tensor imaging in our MRI protocol likely underestimated the amount of DAI present in this cohort. However, this method is novel, and GRE (or susceptibility weighted imaging) and FLAIR are still the most commonly used protocols in order to detect different types of DAI today.

Previously, parts of this cohort have been used to analyze NF-L, S100B, and NSE samples and their correlation to outcome in retrospective studies.^6,11,29^ However, for NF-L, a less-sensitive assay was then used and those results were not included in this study.^84^ We believe that inclusion of the clinically implemented S100B and NSE provides a valuable comparison for the more novel markers. It should also be mentioned that none of these newer markers have rapid clinical assays; thus, it takes hours to analyze them, making them yet more difficult to implement for clinical decision making.

We have, in a previous study, meticulously analyzed the effects of the S100B assay change during the study period, but have not been able to show any difference between the samples acquired before, and after, the implementation of the Roche^®^ Cobas^®^ system.^6^ Presumably, this is attributed to the fact that the variation described between these two platforms are primarily observed at higher concentrations than the ones commonly encountered clinically.^85^

We acknowledge that the CT scoring systems used in this article are only surrogate markers for injury severity. Ideally, volumetric maps of the affected brain areas, in order to more accurately quantify the amount of injured brain, should be compared with protein biomarker levels.

The CVs for NF-L, tau, and GFAP were below or around 10%, which is clearly acceptable and well within what is commonly observed in immunoassays. Although CVs for UCH-L1 were higher, it could have made results more uncertain at the individual sample level, even if we believe that this will be balanced at group level considering the amounts of patient included in this study.

## Conclusions

We found that S100B, UCH-L1, GFAP, and tau provided highly significant prediction of GOS and NF-L that of mortality, following TBI. When adjusting for known important predictors of TBI outcome, GFAP and NF-L in combination were found to add the most significant information to multi-variable prediction models. Biomarkers of similar cellular origin and temporal trajectories display strong intercorrelations and similar PCA projections, suggesting why they do not add significant independent information when combined. The combination of different biomarkers, reflecting different cellular origins and pathophysiological processes, significantly improved the prediction models and should represent a valuable tool for improved patient stratification in future TBI trials.

## Supplementary Material

Supplemental data

Supplemental data

Supplemental data

Supplemental data

Supplemental data

Supplemental data

Supplemental data

Supplemental data

Supplemental data

Supplemental data
