# Supplementary material for: A Serum Protein Biomarker Panel Improves Outcome Prediction in Human Traumatic Brain Injury
Source: J Neurotrauma. 2019 Sep 23;36(20):2850–62. doi: 10.1089/neu.2019.6375 (PMC6761606; doi:10.1089/neu.2019.6375)
Supplement: Supplemental data [file Suppl_TableS1.docx]

Supplementary Table 1 – Additional patient demographics

| **Computerized tomography** | **Subcategory/Units** | **n=172 patients** |
| --- | --- | --- |
| Marshall CT classification | Grade I | 0 |
|  | Grade II | 35 (20%) |
|  | Grade III | 33 (19%) |
|  | Grade IV | 5 (3%) |
|  | Grade V+VI | 99 (58%) |
| Rotterdam CT score | Score 1 | 1 |
|  | Score 2 | 9 |
|  | Score 3 | 47 |
|  | Score 4 | 57 |
|  | Score 5 | 45 |
|  | Score 6 | 13 |
| Stockholm CT score | median (IQR) | 2.5 (2.0 - 3.3) |
| Progression of hemorrhage on second CT | Yes | 47 (27%) |
|  | Missing second CT | 3 |
| **Head MRI (subset of 81 patients)** |  |  |
| Presence of DAI | Yes (% of MRI patients) | 38 (47%) |
| **Injury severity scoring** |  |  |
| Significant multitrauma | Yes | 49 (28%) |
| Head Abbreviated Injury Score (AIS) | Score 1 | 0 |
|  | Score 2 | 1 |
|  | Score 3 | 18 (10%) |
|  | Score 4 | 54 (31%) |
|  | Score 5 | 94 (55%) |
|  | Missing data | 5 |
| Injury Severity Score (ISS) | median (IQR) | 25 (18 - 29) |
|  | Missing data |  |
| New Injury Severity Score (NISS) | median (IQR) | 43 (34 - 57) |
|  | Missing data | 10 |

Additional demographics for the included patients. Abbreviations: CT – Computerized Tomography, MRI – Magnetic Resonance Imaging, DAI – Diffuse Axonal Injury, IQR – Interquartile range.
