# Supplementary material for: A Serum Protein Biomarker Panel Improves Outcome Prediction in Human Traumatic Brain Injury
Source: J Neurotrauma. 2019 Sep 23;36(20):2850–62. doi: 10.1089/neu.2019.6375 (PMC6761606; doi:10.1089/neu.2019.6375)
Supplement: Supplemental data [file Suppl_TableS2.docx]

Supplementary Table 2 – Sampling information

|  | Days from trauma (median/IQR) | Range (days) |
| --- | --- | --- |
| Sample 1 | 3 (2-4) | 1 to 11 |
| Sample 2 | 6 (5-7) | 2 to 15 |
| Sample 3 | 9 (8-10) | 4 to 15 |
|  |  |  |
|  | Proportion of first sample being peak sample |  |
| S100B | 91% |  |
| NSE | 73% |  |
| GFAP | 95% |  |
| UCHL1 | 80% |  |
| Tau | 78% |  |
| NF-L | 20% |  |

Additional information concerning sampling. Abbreviations: IQR – Interquartile range.
