# Supplementary material for: A Serum Protein Biomarker Panel Improves Outcome Prediction in Human Traumatic Brain Injury
Source: J Neurotrauma. 2019 Sep 23;36(20):2850–62. doi: 10.1089/neu.2019.6375 (PMC6761606; doi:10.1089/neu.2019.6375)
Supplement: Supplemental data [file Suppl_FigureS1.pdf]

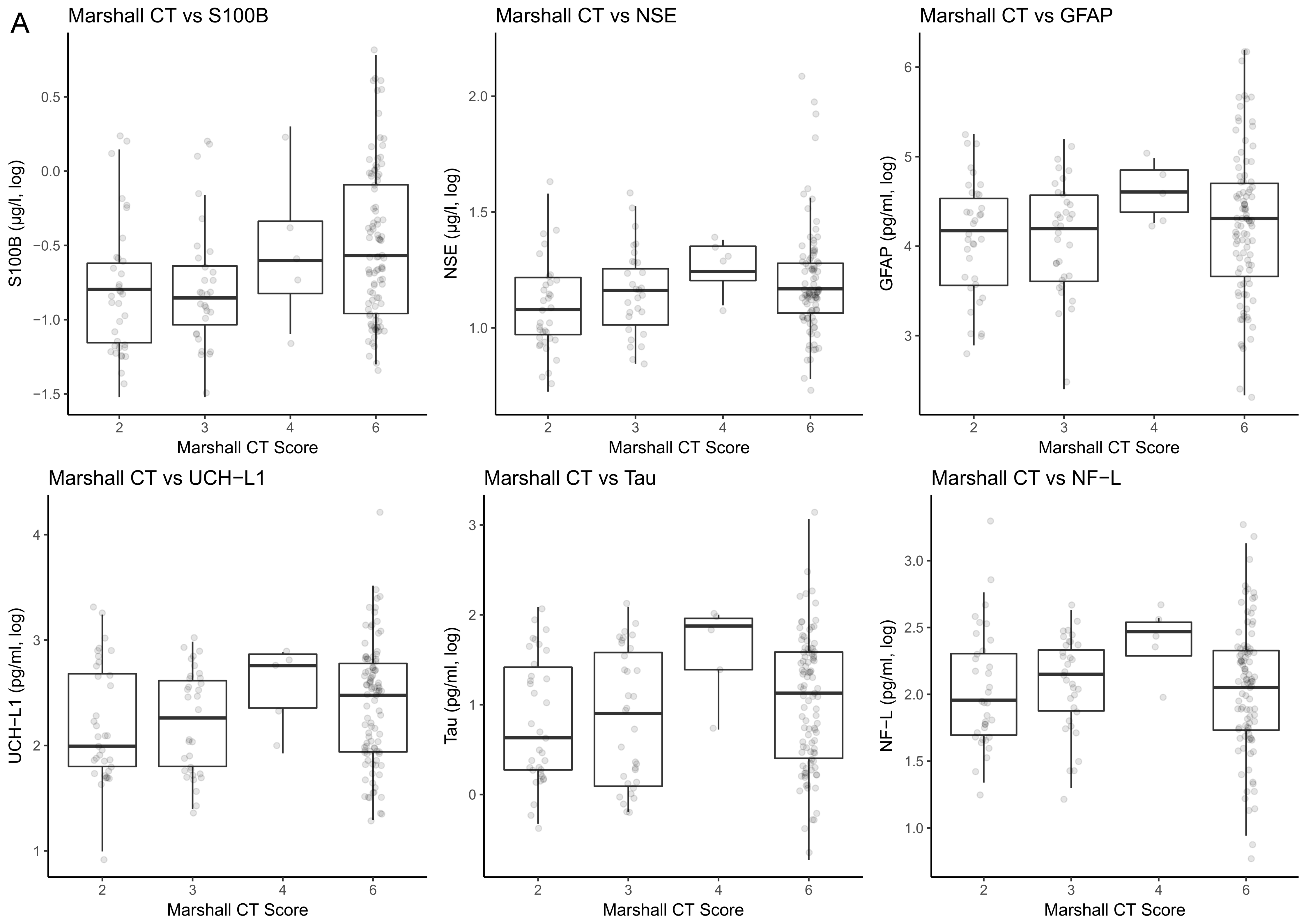
A
Marshall CT vs S100B
S100B (µg/l, log)
Marshall CT Score
Marshall CT vs NSE
NSE (µg/l, log)
Marshall CT Score
Marshall CT vs GFAP
GFAP (pg/ml, log)
Marshall CT Score
Marshall CT vs UCH−L1
UCH−L1 (pg/ml, log)
Marshall CT Score
Marshall CT vs Tau
Tau (pg/ml, log)
Marshall CT Score
Marshall CT vs NF−L
NF−L (pg/ml, log)
Marshall CT Score

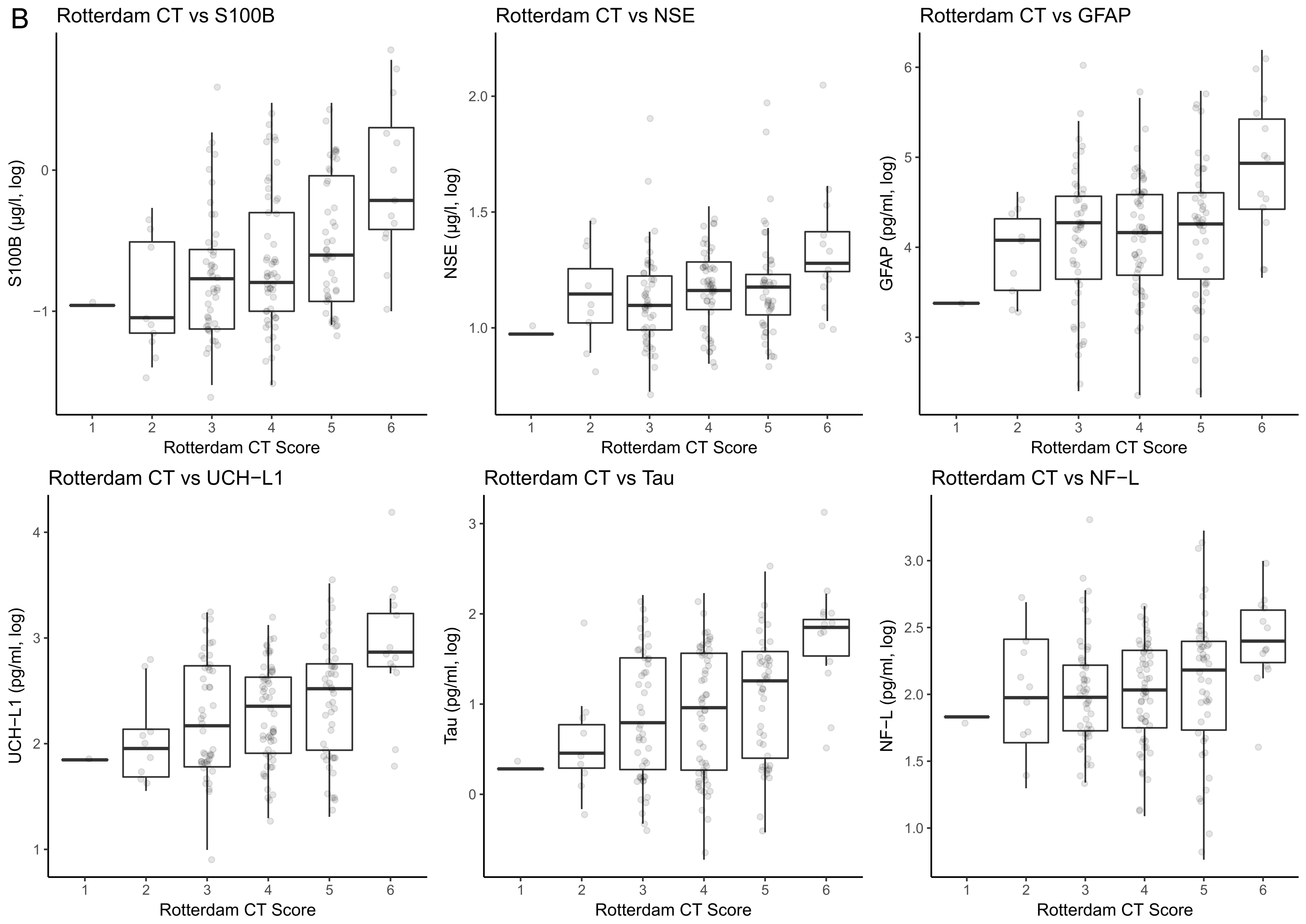

B
Rotterdam CT vs S100B
S100B (µg/l, log)
Rotterdam CT Score
Rotterdam CT vs NSE
NSE (µg/l, log)
Rotterdam CT Score
Rotterdam CT vs GFAP
GFAP (pg/ml, log)
Rotterdam CT Score
Rotterdam CT vs UCH−L1
UCH−L1 (pg/ml, log)
Rotterdam CT Score
Rotterdam CT vs Tau
Tau (pg/ml, log)
Rotterdam CT Score
Rotterdam CT vs NF−L
NF−L (pg/ml, log)
Rotterdam CT Score

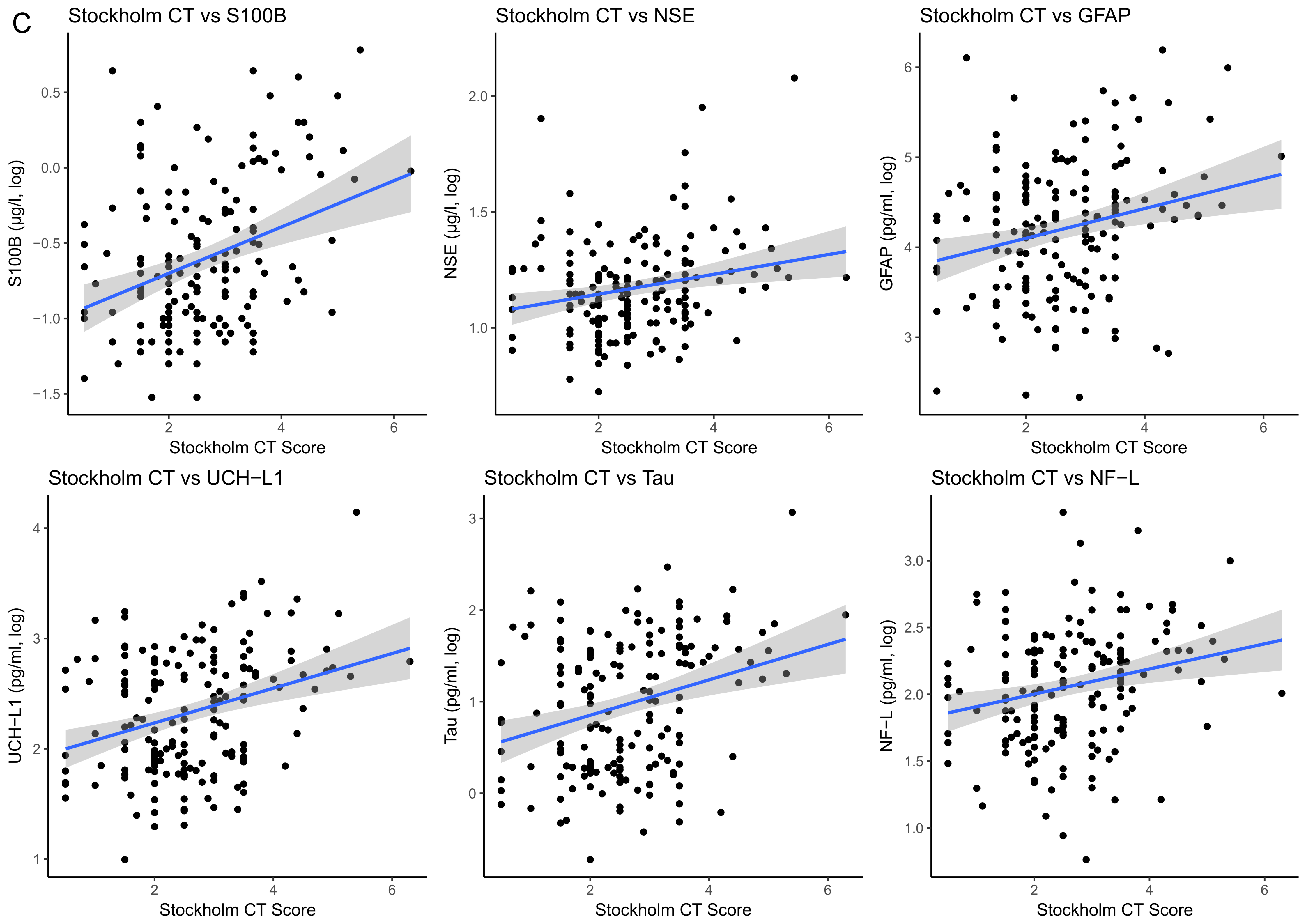
C
Stockholm CT vs S100B
S100B (µg/l, log)
Stockholm CT Score
Stockholm CT vs NSE
NSE (µg/l, log)
Stockholm CT Score
Stockholm CT vs GFAP
GFAP (pg/ml, log)
Stockholm CT Score
Stockholm CT vs UCH−L1
UCH−L1 (pg/ml, log)
Stockholm CT Score
Stockholm CT vs Tau
Tau (pg/ml, log)
Stockholm CT Score
Stockholm CT vs NF−L
NF−L (pg/ml, log)
Stockholm CT Score
