# Supplementary figures and images for: A Serum Protein Biomarker Panel Improves Outcome Prediction in Human Traumatic Brain Injury
Source: J Neurotrauma. 2019 Sep 23;36(20):2850–62. doi: 10.1089/neu.2019.6375 (PMC6761606; doi:10.1089/neu.2019.6375)

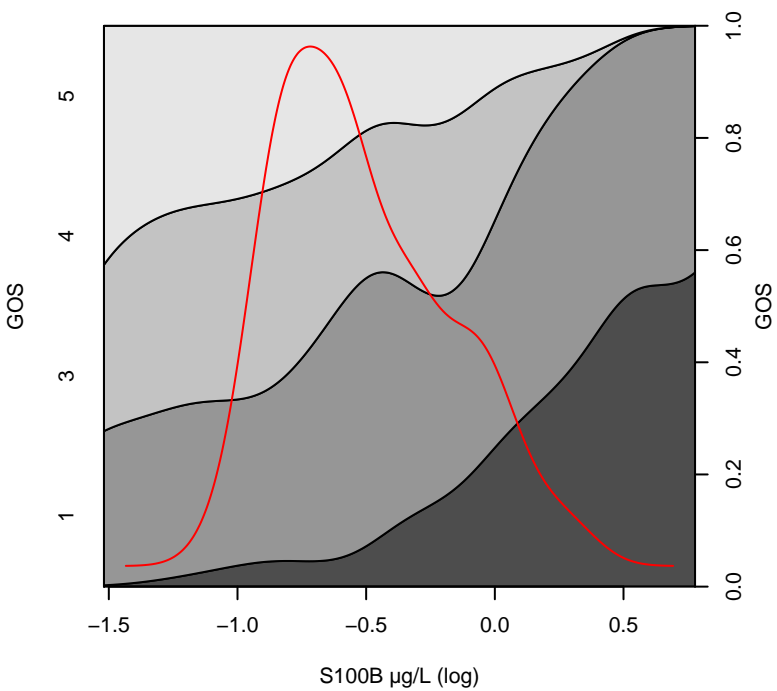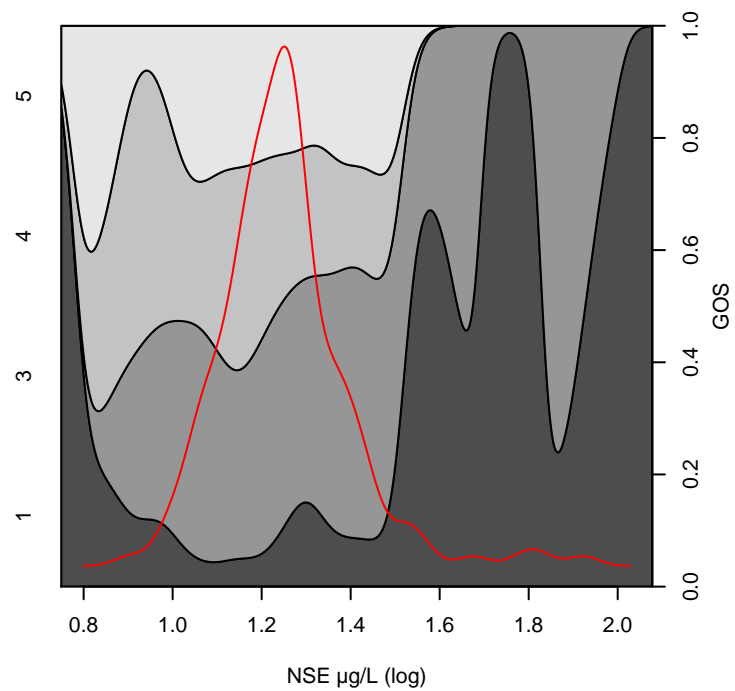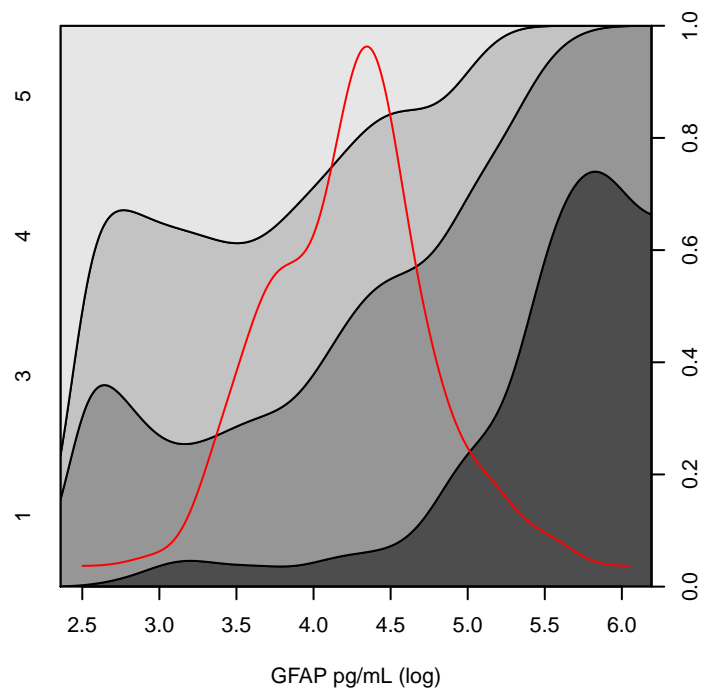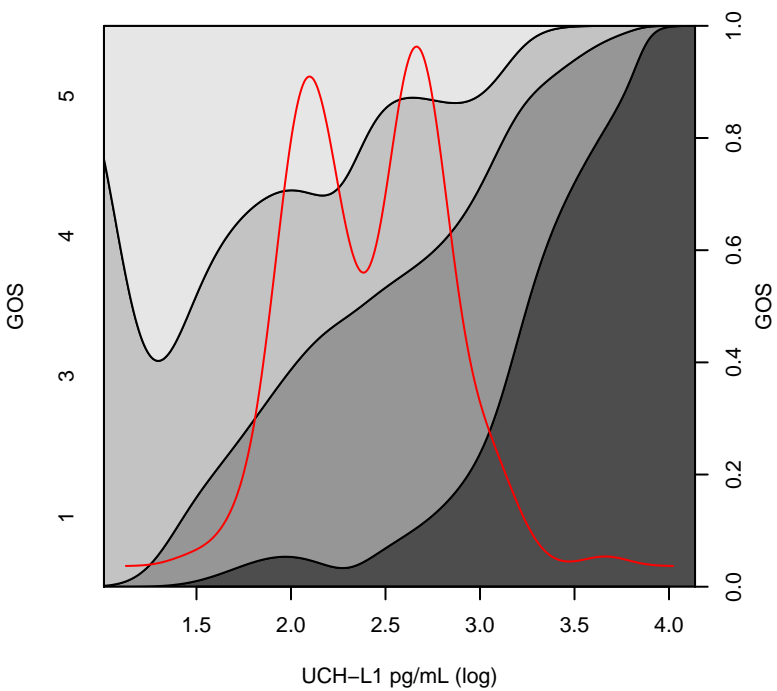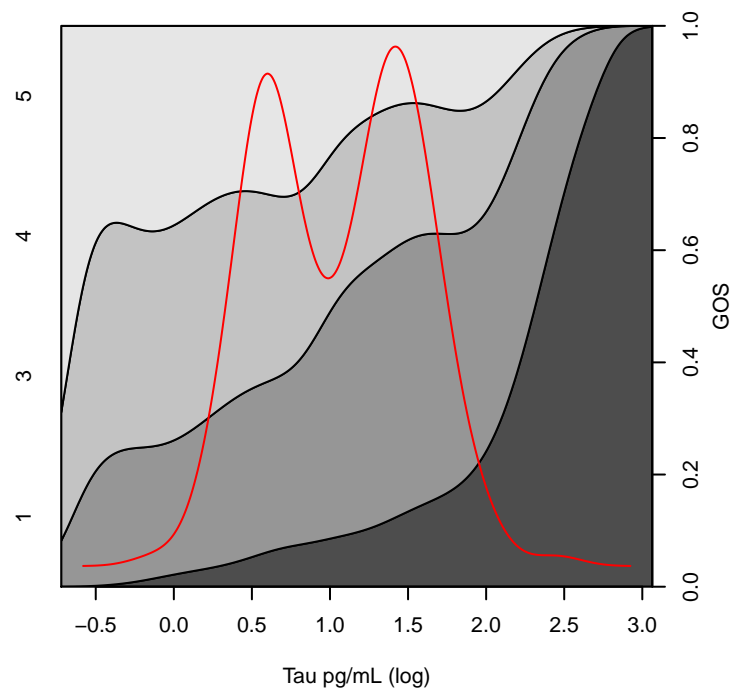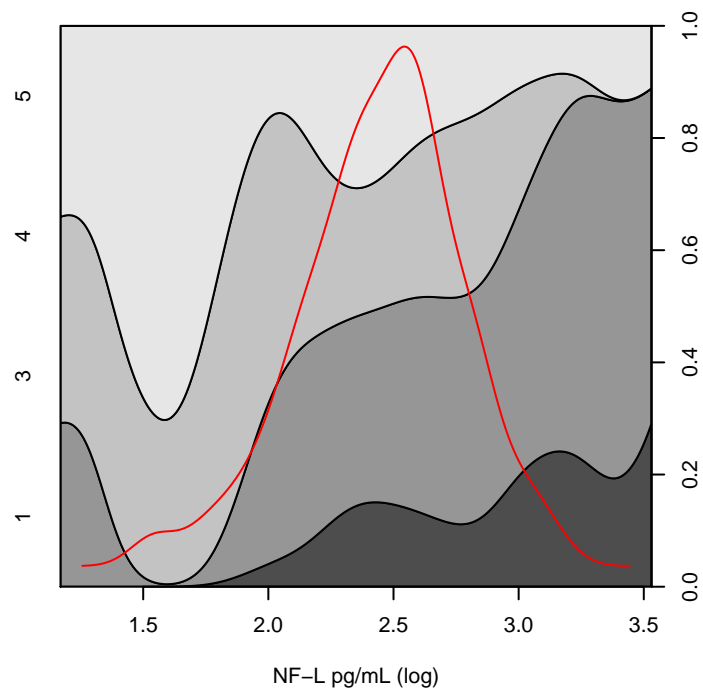

Supplement: Supplemental data [file Suppl_FigureS2.pdf]

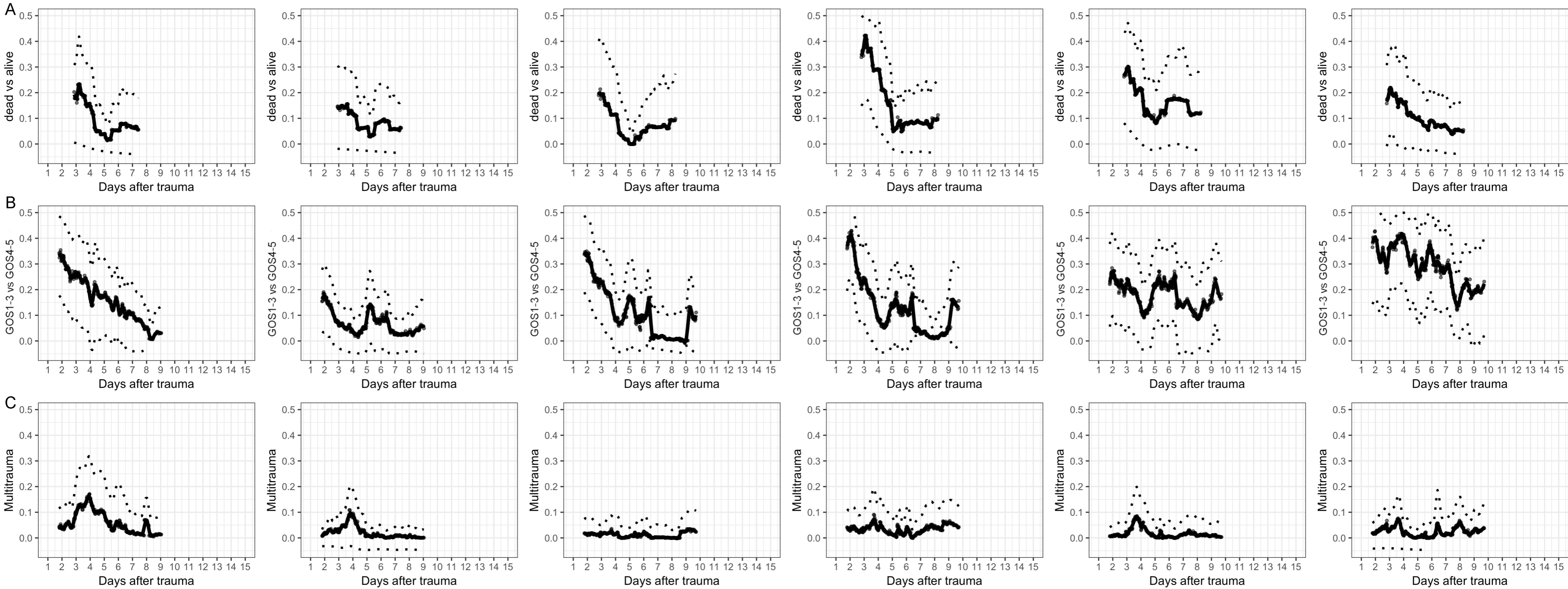

Supplement: Supplemental data [file Suppl_FigureS3.pdf]

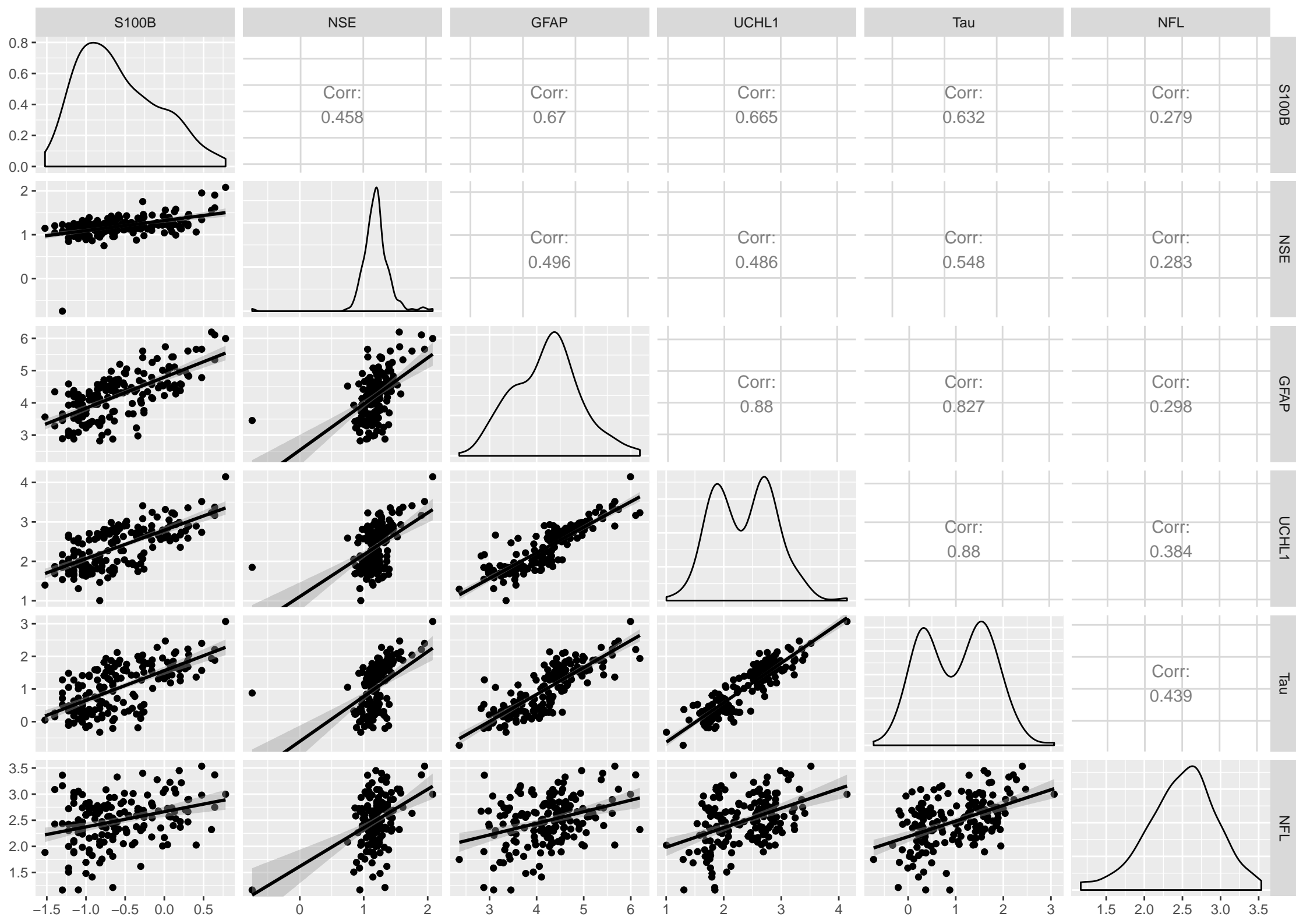

Supplement: Supplemental data [file Suppl_FigureS4.pdf]
