# Supplementary material for: A Serum Protein Biomarker Panel Improves Outcome Prediction in Human Traumatic Brain Injury
Source: J Neurotrauma. 2019 Sep 23;36(20):2850–62. doi: 10.1089/neu.2019.6375 (PMC6761606; doi:10.1089/neu.2019.6375)
Supplement: Supplemental data [file Suppl_TableS3.docx]

Supplementary Table 3 – Biomarker levels versus levels of GOS and number of samples the first 5 days.

| S100B (µg/l) | Day1 | Day2 | Day3 | Day4 | Day5 |
| --- | --- | --- | --- | --- | --- |
| GOS1 | 2.00 (0.81-3.00) | 1.3 (1.12-2.5) | 1.33 (1.02-1.51) | 0.27 (0.13-0.42) | 0.58 (0.36-0.84) |
| GOS3 | 0.33 (0.27-1.0) | 0.22 (0.1-0.51) | 0.26 (0.17-1.4) | 0.15 (0.10-0.42) | 0.09 (0.06-0.23) |
| GOS4 | 0.21 (0.18-0.22) | 0.35 (0.10-0.58) | 0.14 (0.10-0.19) | 0.09 (0.07-0.15) | 0.06 (0.05-0.09) |
| GOS5 | 0.11 (0.10-0.23) | 0.12 (0.08-0.16) | 0.19 (0.09-0.25) | 0.05 (0.05-0.15) | 0.07 (0.05-0.09) |
|  |  |  |  |  |  |
| NSE (µg/l) | Day1 | Day2 | Day3 | Day4 | Day5 |
| GOS1 | 22 (19-26) | 37 (18-73) | 16 (12-24) | 10 (8.0-13) | 15 (11-16) |
| GOS3 | 16 (12-18) | 16 (13-18) | 14 (10-18) | 15 (12-17) | 11 (8.8-17) |
| GOS4 | 13 (11-13) | 14 (12-17) | 13 (11-20) | 14 (10-16) | 11 (8.3-15) |
| GOS5 | 16 (11-19) | 17 (13-18) | 14 (12-18) | 11 (11-13) | 11 (9.9-12) |
|  |  |  |  |  |  |
| GFAP (pg/ml) | Day1 | Day2 | Day3 | Day4 | Day5 |
| GOS1 | 235687 (404485-81062) | 402995 (192799-503070) | 86914 (64289-136957) | 10267 (3737-28225) | 21376 (13457-31976) |
| GOS3 | 40554 (22375-117125) | 22145 (10523-28541) | 35762 (16621-44619) | 21041 (14849-30877) | 4892 (1879-15161) |
| GOS4 | 33646 (25633-34316) | 12128 (2854-33588) | 19079 (11131-37807) | 4167 (1194-8671) | 4643 (2592-13349) |
| GOS5 | 11422 (2850-14880) | 8783 (3511-18924) | 7112 (3633-25835) | 3867 (3747-16879) | 1662 (348-2465) |
|  |  |  |  |  |  |
| UCH-L1 (pg/ml) | Day1 | Day2 | Day3 | Day4 | Day5 |
| GOS1 | 1327 (649-1704) | 2061 (1253-2926) | 891 (755-1120) | 210 (131-351) | 343 (265-431) |
| GOS3 | 628 (262-858) | 381 (133-581) | 373 (185-549) | 245 (164-401) | 207 (63-428) |
| GOS4 | 315 (236-371) | 100 (58-458) | 226 (75-409) | 73 (45-196) | 99 (58-278) |
| GOS5 | 134 (70-185) | 101 (59-177) | 79 (48-193) | 139 (35-227) | 50 (30-64) |
|  |  |  |  |  |  |
| Tau (pg/ml) | Day1 | Day2 | Day3 | Day4 | Day5 |
| GOS1 | 86 (36-167) | 80 (73-202) | 42 (38-62) | 12 (5.4-57) | 38 (35-64) |
| GOS3 | 30 (12-38) | 17 (2.1-36) | 38 (13-51) | 12 (3.4-23) | 9.5 (2.9-31) |
| GOS4 | 14 (5.8-25) | 2.3 (1.9-32) | 9.1 (2.1-28) | 3.2 (1.2-12) | 6.6 (1.2-18) |
| GOS5 | 7.5 (1.6-21) | 2.6 (1.9-6.2) | 2.8 (1.2-10) | 2.4 (1.9-21) | 0.73 (0.67-0.86) |
|  |  |  |  |  |  |
| NF-L (pg/ml) | Day1 | Day2 | Day3 | Day4 | Day5 |
| GOS1 | 210 (140-347) | 294 (242-713) | 205 (181-306) | 195 (143-497) | 254 (190-480) |
| GOS3 | 126 (100-194) | 109 (49-204) | 262 (154-428) | 167 (107-269) | 245 (186-429) |
| GOS4 | 56 (30-91) | 54 (42-103) | 94 (49-165) | 60 (45-107) | 107 (90-144) |
| GOS5 | 37 (19-41) | 61 (31-100) | 61 (50-113) | 79 (71-109) | 53 (49-95) |
|  |  |  |  |  |  |
| Sample numbers |  |  |  |  |  |
| GOS1 | n=5 | n=7 | n=4 | n=6 | n=4 (3 for S100B/NSE) |
| GOS3 | n=12 | n=18 (17 for S100B/NSE) | n=13 (12 for S100B/NSE) | n=16 (15 for S100B/NSE) | n=17 |
| GOS4 | n=4 | n=19 | n=11 | n=8 (7 for S100B/NSE) | n=13 (11 for S100B/NSE) |
| GOS5 | n=9 | n=10 | n=12 (11 for S100B/NSE) | n=5 | n=5 (4 for S100B and 2 for NSE) |

Supplementary Table 3 illustrates the biomarker levels versus levels of GOS the first 5 days number of samples per day and different GOS levels, as well as number of samples per day for different GOS. Abbreviations: GOS – Glasgow Outcome Scale
