# Supplementary material for: A Serum Protein Biomarker Panel Improves Outcome Prediction in Human Traumatic Brain Injury
Source: J Neurotrauma. 2019 Sep 23;36(20):2850–62. doi: 10.1089/neu.2019.6375 (PMC6761606; doi:10.1089/neu.2019.6375)
Supplement: Supplemental data [file Suppl_TableS4.docx]

Supplementary Table 4 – Multivariable analyses versus patient outcome using an ordinal regression of GOS

| **GOS 1 vs 3 vs 4 vs 5 (proportional odds)** | | |
| --- | --- | --- |
| IMPACT + Rotterdam CT score | 0.275 |  |
| Base (IMPACT + Stockholm CT score) | 0.352 |  |
| Base + S100B | 0.430* (p<0.001) |  |
| Base + NSE | 0.395* (p=0.001) |  |
| **Base + UCH-L1:** | **0.472*** (p<0.001) |  |
| Base + Tau: | 0.458* (p<0.001) |  |
| Base + GFAP: | 0.436* (p<0.001) |  |
| Base + NF-L: | 0.394* (p=0.001) |  |
| Base + UCH-L1 + S100B: | 0.478 |  |
| Base + UCH-L1 + NSE: | 0.473 |  |
| Base + UCH-L1 + GFAP: | 0.478 |  |
| Base + UCH-L1 + Tau: | 0.481 |  |
| **Base + UCH-L1 + NF-L:** | **0.482 (p=0.08)** |  |
| **Base + UCH-L1 + NF-L + S100B:** | **0.487** |  |
| Base + UCH-L1 + NF-L + NSE: | 0.484 |  |
| **Base + UCH-L1 + NF-L + GFAP:** | **0.487** |  |
| Base + UCH-L1 + NF-L + Tau: | 0.486 |  |

Multivariable regression analyses versus GOS 1 vs 3 vs 4 vs 5 at 12 months using ordinal regression analysis. The IMPACT model consists of age, GCS, pupil response, scene of accident hypoxia, scene of accident hypotension, admission glucose and admission hemoglobin. To this, Rotterdam CT score was added initially, but then replaced by Stockholm CT-score forming the “Base” model used. * = step-up model significantly improved compared to the Base model. ‡ = step-up model significantly improved compared to Base + UCH-L1 model. Abbreviations: IMPACT - International Mission for Prognosis and Analysis of Clinical Trials in TBI, CT – Computerized Tomography, GOS – Glasgow Outcome Scale.
