# Supplementary material for: A Serum Protein Biomarker Panel Improves Outcome Prediction in Human Traumatic Brain Injury
Source: J Neurotrauma. 2019 Sep 23;36(20):2850–62. doi: 10.1089/neu.2019.6375 (PMC6761606; doi:10.1089/neu.2019.6375)
Supplement: Supplemental data [file Suppl_TableS5.docx]

Supplementary Table 5 – Principal Component Analysis of the protein biomarkers’ peak concentration

| Principal component | Eigenvalue | Variance (%) | Cumulative variance (percent) |
| --- | --- | --- | --- |
| 1 | 4.041 | 67.343 | 67.343 |
| 2 | 0.869 | 14.480 | 81.823 |
| 3 | 0.599 | 9.989 | 91.814 |
| 4 | 0.251 | 4.187 | 96 |
| 5 | 0.201 | 3.350 | 99.350 |
| 6 | 0.039 | 0.650 | 100.00000 |

Principal component analysis (PCA) performed on the peak concentrations from each patient. The table lists the outputs from the PCA components. The eigenvalue and variance for each component, as well as the added up cumulative variance, are described in the columns.
